# Remodeling of Autogenous Bone Grafts after Osteotome Sinus Floor Elevation Assessed by Limited Cone Beam Computed Tomography

**DOI:** 10.1155/2013/931708

**Published:** 2013-07-14

**Authors:** Tetsuya Nishida, Yuka Takenouchi, Kyoko Mori, Miyuki Ariji, Kaori Nishida, Koichi Ito

**Affiliations:** ^1^Department of Periodontology, Nihon University School of Dentistry, 1-8-13 Kanda-Surugadai, Chiyoda-ku, Tokyo 101-8310, Japan; ^2^Division of Advanced Dental Treatment, Dental Research Center, Nihon University School of Dentistry, 1-8-13 Kanda-Surugadai, Chiyoda-ku, Tokyo 101-8310, Japan; ^3^Ark Dental Clinic, 1-5 Rokubancho, Chiyoda-ku, Tokyo 102-0085, Japan; ^4^Nihon University School of Dentistry, 1-8-13 Kanda-Surugadai, Chiyoda-ku, Tokyo 101-8310, Japan

## Abstract

This study assessed the radiographic appearance of bone graft domes longitudinally after osteotome sinus floor elevation using cone beam computed tomography (CBCT). This study presents the radiological findings of a 6-month follow-up CBCT study in maxillary osteotome sinus floor elevation. We examined 52 patients with a crestal bone height of less than 8 mm in the posterior maxilla who required sinus augmentation. Implants (*n* = 91) were subsequently placed in regenerated bone following osteotome sinus floor elevation; autogenous bone was used as the augmentation material. In all cases, the grafted augmentation material tended to be absorbed, but at least 1 mm of grafted augmentation material was recognized around the implant fixtures on CBCT at the second implant operation. The border between the grafted augmentation material and the existing bone was indistinct. The grafted area apical to the implants undergoes shrinkage and remodeling. It was suggested that sufficient grafted autogenous bone changes into bone to support an implant.

## 1. Introduction

Alveolar bone resorption of the maxillary posterior edentulous region and increased pneumatization of the sinus cavity can result in insufficient bone support for dental implants. This problem can be overcome by grafting the maxillary sinus floor using a sinus lift procedure [1–12]. A prerequisite for this procedure is separating the intact sinus membrane from the maxillary sinus floor. The periosteal portion of this membrane has few elastic fibers, making the separation a relatively simple and reliable procedure. As a result, the posterior maxilla is one of the most predictably successful areas for bone grafting procedures [13]. Numerous articles describe this procedure detailing bone grafting materials, long-term clinical follow-up of bone consolidation, and implant success [1–12]. Complications following a sinus lift procedure include maxillary sinusitis, oroantral communication, bone graft resorption, mucocele formation, maxillary cyst, and implant failure [14]. 

Currently, two main approaches to the maxillary sinus floor elevation procedure can be found in the literature. The first approach, lateral antrostomy, is the classical and more commonly performed technique, originally described by Tatum [15]. The lateral antrostomy technique, also referred to as the Caldwell-Luc or lateral window approach, involves creating a window in the lateral wall of the maxillary sinus to permit visualization of the Schneiderian membrane during its elevation in preparation for site grafting. More recently, Summers advocated a second approach, the crestal approach, using osteotomes [16–18]. The osteotome technique takes advantage of the fact that bone is viscoelastic and can be compressed and manipulated. Bone compaction, cortical sinus floor elevation, and ridge expansion can be performed using the technique, which has been evaluated in several clinical studies [19–25]. 

Recently, a new compact computed tomography (CT) system, known as ortho cubic super-high-resolution CT, was developed. Limited cone beam CT (CBCT) provides three-dimensional (3D) information. A restriction with conventional radiographic methods is the limited two-dimensional (2D) information they provide. For the complete assessment of the grafted dome-shaped bone graft after an osteotomy, a 3D evaluation method is required. The aim of this study was to longitudinally assess the radiographically appearing bone graft domes after osteotomy using CBCT. 

## 2. Material and Methods

### 2.1. Patient Selection

All patients with a crestal bone height of less than 8 mm in the posterior maxilla who required sinus augmentation were chosen from those seen at Nihon University School of Dentistry Dental Hospital, Tokyo, Japan, after evaluating their medical histories and conducting thorough dental examinations between February 2003 and July 2011. 

### 2.2. Sinus Lift Procedure Technique

**Figure 1:**
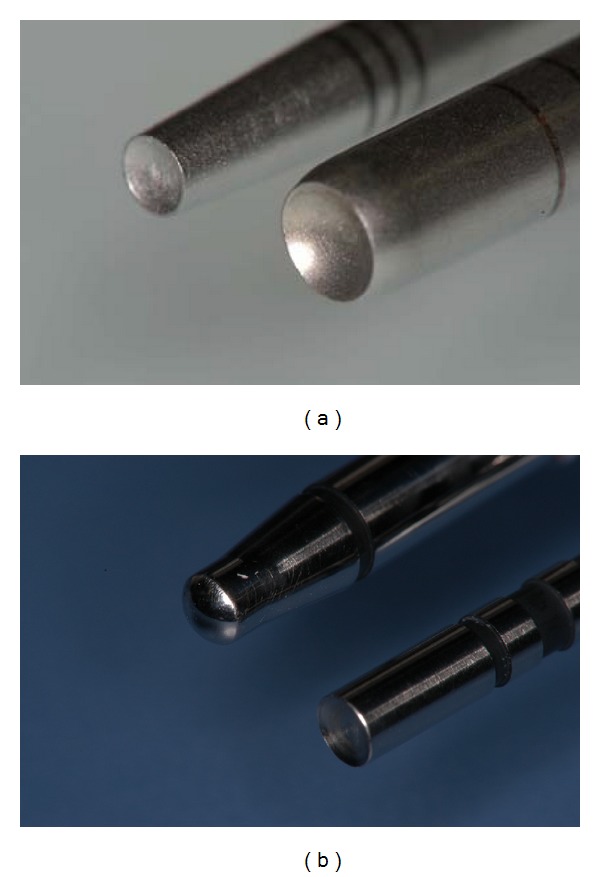
The osteotomes used for the sinus floor elevation. Summers' (a) and N-type (b) osteotomes.

The osteotome sinus floor elevation procedure was performed as described by Summers [16–18], and autogenous bone was used as the augmentation material. The grafted autogenous bone was collected in the local bone of the implant placement and other areas. A crestal incision was made into the molar regions. Detachment of vestibular and palatal mucoperiosteal flaps revealed the top of the bony crest. In these operations, two kinds of instrument were used: a Summers' osteotome and the original instrument, referred to as an N-type osteotome (Figure 1). Implants (ASTRA; Astra Tech AB, Molndal, Sweden), 5.0 mm in diameter and 11.0 mm long, were placed in the bone regenerated following osteotome sinus floor elevation. The mucoperiosteal flap was closed over the graft and the implants using 5-0 sutures, and the patients were not permitted to wear their dentures for the first 2 postoperative weeks. Second-stage surgery was carried out 6 months after implant placement. The patients were evaluated radiographically before implant exposure. CBCT was used to assess the newly formed bone and its interface with the implants, the condition of the sinus membrane, and the presence of any sinus pathology. 

### 2.3. CBCT Observations

**Figure 2:**
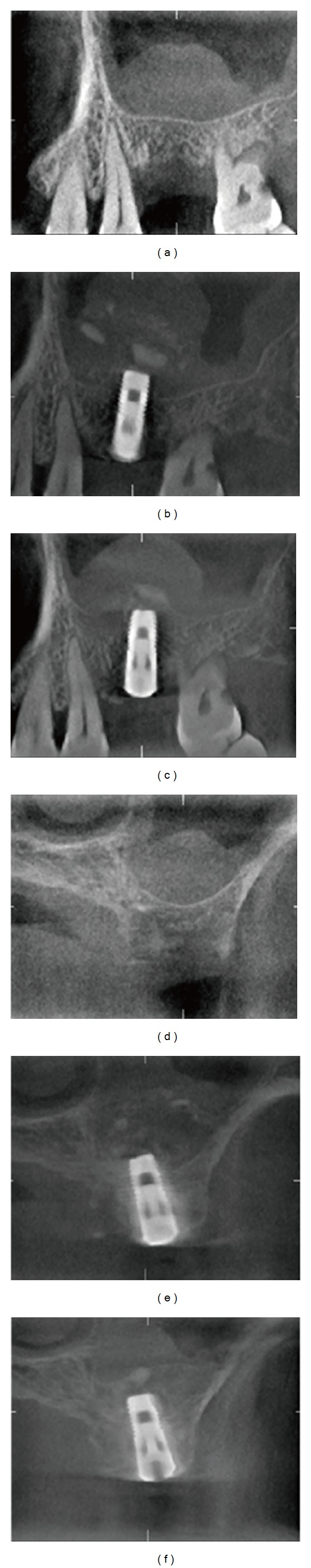
Representative CBCT images. Parallel section to dental arch (a) preoperatively and at the first (b) and second (c) operations. Cross-section to dental arch (d) preoperatively and at the first (e) and second (f) operations.

**Figure 3:**
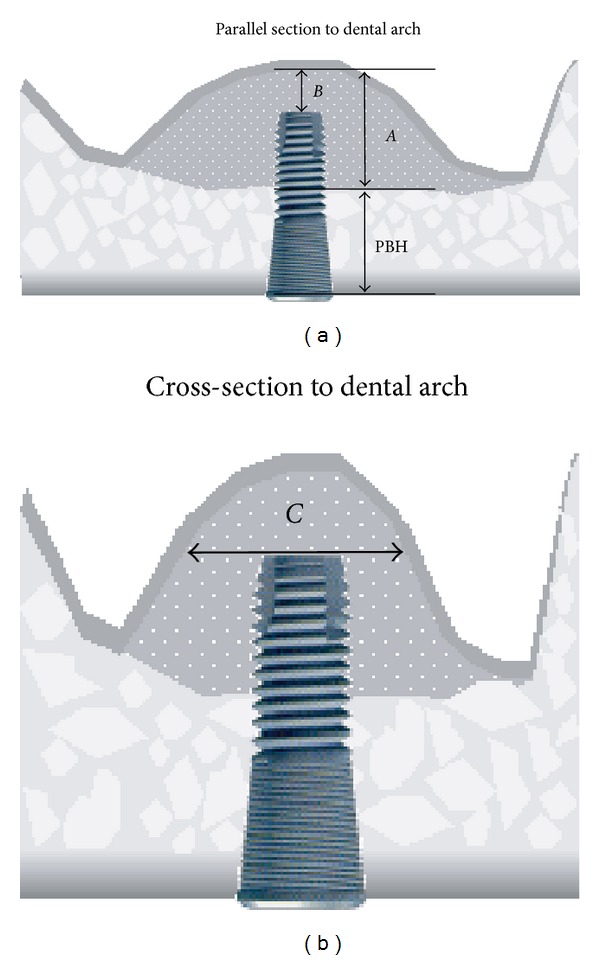
Schematic of the measurement points on a CBCT images PBH: preoperative crestal bone height, (*A*) total height of the bone graft, (*B*) apical height of the bone graft, and (*C*) width of the bone graft.

The CBCT system was used to observe the change in grafted autogenous bone after osteotome sinus floor elevation. CBCT was performed before (Figures 2(a) and 2(d)) and just after (Figures 2(b) and 2(e)) the first implant operation and at the second implant operation (Figures 2(c) and 2(f)). The preoperative crestal bone height (PBH) and total height (*A*), local height (*B*), and width (*C*) of the sinus floor elevation were measured on CBCT at the first and second operations (Figure 3).

The measurement of a sample on CBCT image was performed using the measurement tool available on the proprietary software (i-VIEW One Volume Viewer Ver. 1.5.0, Morita Co., Kyoto, Japan) by three observers who were dentists with clinical experience in periodontics dentistry of more than 5 years to adjust the measurement error.

## 3. Results

We examined 52 patients in the posterior maxilla who required sinus augmentation. Their ages ranged from 30 to 64 years (mean 49.1 years). Implants (*n* = 91) were subsequently placed in regenerated bone following osteotome sinus floor elevation; autogenous bone was used as the augmentation material. 

Most of the patients experienced no severe pain, swelling, or nose bleed after the first implant operation, although one patient had a slight nose bleed 2 days after the surgery. The grafted autogenous bone was in the shape of a dome around the fixtures on the CBCT image at the first operation. 

**Figure 4:**
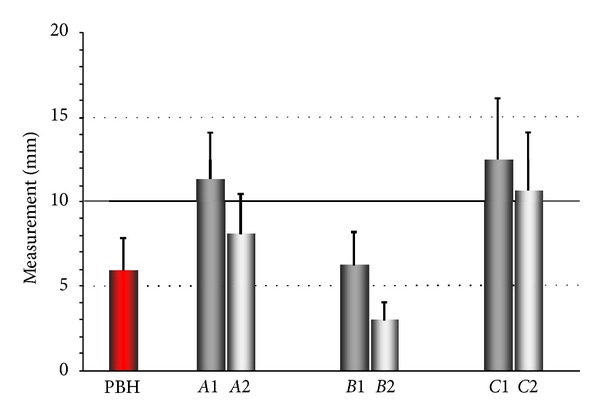
The average measurements on a CBCT image. PBH: preoperative crestal bone height; *A*: total height of the sinus floor elevation at the first (1) and second (2) operations; *B*: local height of the sinus floor elevation at the first (1) and second (2) operation; *C*: width of the sinus floor elevation at the first (1) and second (2) operations.

The preoperative crestal bone height (PBH), measured on original CT images, ranged between 3.0 and 8.0 mm, with a mean of 5.9 ± 1.9 mm. The distance (*A*) from the implant shoulder to the grafting material/new bone just after implant placement (*A1*), as measured on original CT images, ranged between 7.3 and 16.5 mm, with a mean of 11.3 ± 2.8 mm; after 6 months of implant placement (*A2*), as measured on original CT images, it ranged between 4.2 and 12.3 mm, with a mean of 8.1 ± 2.4 mm. The distance (*B*) from the apex of the implant to the grafting material/new bone just after implant placement (*B1*), as measured on original CT images, ranged between 4.2 and 8.5 mm, with a mean of 6.3 ± 2.0 mm; after 6 months of implant placement (*B2*), as measured on original CT images, it ranged between 1.0 and 4.5 mm, with a mean of 3.0 ± 1.1 mm. The distance (*C*) from the facial sinus wall to the palatal sinus wall at the height of the apex of the implant just after implant placement (*C1*), as measured on original CT scans, ranged between 6.1 and 22.2 mm, with a mean of 12.4 ± 3.7 mm; after 6 months of implant placement (*C2*), as measured on the original CT scans, it ranged between 6.0 and 16.2 mm, with a mean of 10.7 ± 3.4 mm (Figure 4). The differences between the measurements by the three observers are shown in Table 1.

Second-stage surgery was carried out 6 months after implant placement. All implants achieved good integration at second-stage surgery.

## 4. Discussion

Clinical judgment and radiologic imaging are the standard routine assessment and examination tools, which help determine case prognosis and the adequacy of the surgical results. Using CT to analyze the three-dimensional anatomic planes has become the “gold standard” for determining a comprehensive implant treatment plan and assessing the cancellous and cortical bone postoperatively [26]. To our knowledge, no follow-up study based on CBCT has compared the pre- and postoperative radiologic findings in sinus lift procedures using an osteotome. Brägger et al. assessed the remodeling extensively, but only in two dimensions [27]. 

The CBCT system has a two-dimensional sensor and uses a cone-shaped X-ray beam in place of a fan-shaped one. The machine can acquire volume data in a single rotation of the beam and sensor, is less expensive, and has a higher resolution in the axial plane than a conventional CT system. The risk of radiation exposure is a problem with the three-dimensional CT technique. The radiation dosage from CT has been reported to be much greater than that from panoramic radiographs or intraoral radiographs used for assessing dental implant surgery. The skin dose from CBCT is similar to that from rotation panoramic radiography and several dozen times lower than that from conventional CT of the jaws. The Department of Radiology at Nihon University School of Dentistry has developed a compact CBCT apparatus for use in dental practice [28]. 

Due to its high resolution and low radiation dose [29], this CBCT has been proven to be useful for preoperative examinations before minor oral surgery and implant surgery [30, 31]. 

The goal in treating the severely atrophic maxilla is to provide the patient with an implant-supported prosthesis that will be successful in the long-term and will result in minimal adverse reactions. 

Rosen et al. [23] reported data from a retrospective analysis of 174 implants was placed in 101 patients using Summers' osteotome technique [16–18]; the average period of implant loading was 20.2 months, with a range of 6 to 66 months. The survival rate was 96% or higher when the pretreatment bone height was 5 mm or more and dropped to 85.7% when the pretreatment bone height was 4 mm or less. Cavicchia et al. [32] reported a survival rate of 88.6% after a mean observation period of 35 months for 97 implants placed using Summers' crestal access method [16–18]. A recent study reported an overall survival rate of 93.5% for 276 implants loaded for an average of 27.9 months [33]; similarly, in other studies, when only sites with a residual bone height of the alveolar ridge of 4 mm or less were considered, the survival rate dropped to 73.3%. From the analysis of the previously cited works and other recently published studies [34, 35], one of the most important factors influencing implant survival with osteotome sinus floor elevation is the preexisting bone height between the sinus floor and crest. A residual bone height equal to or less than 4 mm is associated with reduced primary implant stability because the graft material does not provide immediate support for the implant [36–39]. A finite element analysis has shown that reduced augmentation volume, as should be expected with osteotome sinus floor elevation, leads to a further decrease in implant stability [40]. In a retrospective study, Li reported that if primary stability can be achieved, osteotome sinus floor elevation can be applied for a residual ridge of 3 to 4 mm in height [41]. 

The grafted autogenous bone was in the shape of a dome around the fixtures on the CBCT image at the first operation. In all cases, the grafted augmentation material tended to be absorbed, but at least 1 mm of grafted augmentation material was seen around the implant fixtures on CBCT at the second implant operation. The border between the grafted augmentation material and existing bone was indistinct. The results show that in areas with reduced bone height subjacent to the sinus, an osteotome technique may provide a minimally invasive way to obtain implant abutments predictably. The grafted area apical to the implants undergoes shrinkage and remodeling, and, thus, the original boundary of the sinus is eventually consolidated and replaced by a new cortical plate. 

## 5. Conclusion

In conclusion, the findings suggest that sufficient grafted autogenous bone changes into bone to support an implant. Therefore, CBCT is effective in osteotome sinus floor elevation for subsequent implant placement. Sinus floor elevation using osteotomes is a useful technique when insufficient crestal bone height exists in the posterior maxilla. Long-term radiographic and clinical observations may be necessary. 

## Figures and Tables

**Table 1:** Measurements on CBCT images of the observers.

Observers	PBH			*A*1			*A*2			*B*1			*B*2			*C*1			*C*2		
	1	2	3	1	2	3	1	2	3	1	2	3	1	2	3	1	2	3	1	2	3
Mean	5.9	5.9	5.9	11.3	11.4	11.3	8.1	8.0	8.1	6.2	6.3	6.3	3.0	3.0	3.1	12.4	12.5	12.5	10.7	10.6	10.7
SD	1.9	1.9	1.9	2.7	2.8	2.8	2.4	2.4	2.4	1.9	2.0	2.0	1.1	1.0	1.1	3.7	3.7	3.7	3.5	3.4	3.4
Min	2.9	3.0	3.0	7.4	7.2	7.1	4.2	4.1	4.2	4.2	4.1	4.2	1.0	1.0	1.1	6.0	6.1	6.0	5.8	6.0	5.9
Max	8.0	8.1	8.3	16.5	16.5	16.5	12.3	12.4	12.6	8.5	8.5	8.5	4.5	4.6	4.9	22.2	22.1	22.3	16.3	16.3	16.3
